# Animal Model of Severe Fever With Thrombocytopenia Syndrome Virus Infection

**DOI:** 10.3389/fmicb.2021.797189

**Published:** 2022-01-11

**Authors:** Jiawen Sun, Yuan-Qin Min, Yunjie Li, Xiulian Sun, Fei Deng, Hualin Wang, Yun-Jia Ning

**Affiliations:** ^1^State Key Laboratory of Virology and National Virus Resource Center, Wuhan Institute of Virology, Chinese Academy of Sciences, Wuhan, China; ^2^Savaid Medical School, University of Chinese Academy of Sciences, Beijing, China; ^3^Center for Biosafety Mega-Science, Chinese Academy of Sciences, Wuhan, China

**Keywords:** severe fever with thrombocytopenia syndrome virus (SFTSV), animal model, emerging infectious disease, bunyavirus, prevention and treatment, viral pathogenesis

## Abstract

Severe fever with thrombocytopenia syndrome (SFTS), an emerging life-threatening infectious disease caused by SFTS bunyavirus (SFTSV; genus *Bandavirus*, family *Phenuiviridae*, order *Bunyavirales*), has been a significant medical problem. Currently, there are no licensed vaccines or specific therapeutic agents available and the viral pathogenesis remains largely unclear. Developing appropriate animal models capable of recapitulating SFTSV infection in humans is crucial for both the study of the viral pathogenic processes and the development of treatment and prevention strategies. Here, we review the current progress in animal models for SFTSV infection by summarizing susceptibility of various potential animal models to SFTSV challenge and the clinical manifestations and histopathological changes in these models. Together with exemplification of studies on SFTSV molecular mechanisms, vaccine candidates, and antiviral drugs, in which animal infection models are utilized, the strengths and limitations of the existing SFTSV animal models and some important directions for future research are also discussed. Further exploration and optimization of SFTSV animal models and the corresponding experimental methods will be undoubtedly valuable for elucidating the viral infection and pathogenesis and evaluating vaccines and antiviral therapies.

## Introduction

Severe fever with thrombocytopenia syndrome (SFTS) as an emerging tick-borne infectious disease is an enormous threat to human life and health due to its high case fatality rates of 12–30% and the transmission routes of not only tick bites but also human-to-human and animal-to-human contacts (Bao et al., 2011; Xu et al., 2011; Yu et al., 2011; Zhang et al., 2011; Jung et al., 2019; Kang et al., 2019; Park E. S. et al., 2019; Yamanaka et al., 2020; Ando et al., 2021; Tsuru et al., 2021). SFTS is characterized by fever, thrombocytopenia, leukocytopenia, hemorrhage, and gastrointestinal symptoms (Yu et al., 2011). As reported, several patients with contagious hemorrhagic fever of unknown cause in Henan and Hubei provinces of China between 2007 and 2009 were firstly identified as SFTS cases and the agents isolated from the serum samples were known as severe fever with thrombocytopenia syndrome virus (SFTSV) (Xu et al., 2011; Yu et al., 2011; Zhang et al., 2011). Since then, SFTS has been found to be mainly prevalent in China, Japan, and South Korea, and there have been sporadic cases reported in Vietnam and Myanmar (Liu Q. et al., 2014; Takahashi et al., 2014; Yun et al., 2014; Tran et al., 2019; He et al., 2020; Lin et al., 2020; Win et al., 2020). Moreover, similar cases caused by the Heartland virus, another emerging pathogen that is genetically closely related to SFTSV, were found in North America, suggesting that SFTS and SFTS-like diseases may have worldwide dissemination (McMullan et al., 2012).

Severe fever with thrombocytopenia syndrome virus is a member of *Bandavirus* genus, *Phenuiviridae* family, *Bunyavirales* order, containing three single-stranded negative-sense genomic RNA segments, i.e., the large (L), medium (M), and small (S) segments (Yu et al., 2011; Kuhn et al., 2020). The L segment encodes the RNA-dependent RNA polymerase (RdRp) associated with the viral ribonucleoprotein (RNP) and catalyzing RNA synthesis (Yu et al., 2011; Vogel et al., 2020; Wang et al., 2020). The M segment encodes the glycoprotein precursor that is further cleaved into Gn and Gc subunits. The S segment encodes the nucleocapsid protein (N) that encapsidates viral RNAs into RNPs (Sun et al., 2018). Moreover, by an ambisense strategy, bandavirus S segment also encodes a non-structural protein (NSs) which is involved in the formation of viral inclusion body and plays important roles at the interface of virus-host interactions (Yu et al., 2011; Ning et al., 2014). Particularly, previous studies by us and others have suggested that NSs hijacks several critical host signaling proteins including kinases TANK-binding kinase 1 (TBK1)/IκB kinase ε (IKKε) and transcription factors signal transducer and activator of transcription 1 (STAT1)/STAT2 into the viral inclusion body “jail,” thus blocking interferon (IFN) antiviral immune responses (Yu et al., 2011; Ning et al., 2014, 2015, 2017, 2019; Santiago et al., 2014; Wu et al., 2014; Feng et al., 2019; Min et al., 2020a,b).

**FIGURE 1:**
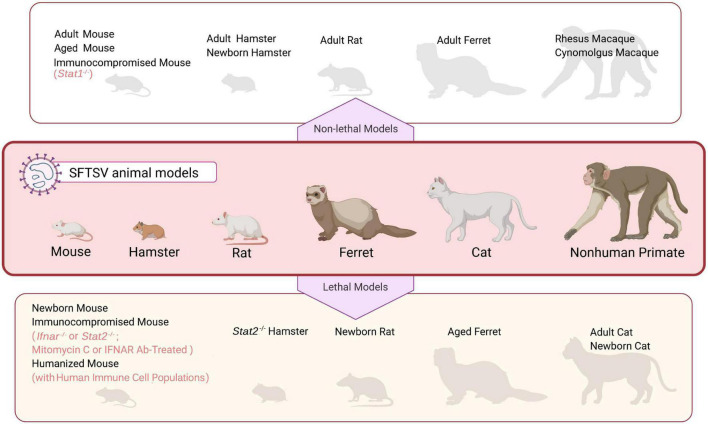
An overview of SFTSV animal models. Mammalian species that have been used in SFTSV research are illustrated in the middle panel. These animal models can be grouped into two categories, “non-lethal models” (upper panel) and “lethal models” (bottom panel), depending on whether SFTSV infection causes death in the animals. See text for details.

Currently, no approved anti-SFTSV drugs or vaccines are available and the mechanisms of pathogenicity and lethality of SFTSV remain to be resolved. To tackle these issues, SFTSV animal models that are able to replicate principal aspects of patients are necessary. Presently, existing animal models devoted to SFTSV researches include various rodents, ferrets, and non-human primates as well as cats (as summarized in Figure 1). Although SFTSV infects a wide range of animal species in nature, most immunocompetent animals tested are insusceptible to SFTSV and have no or only mild symptoms (with the exception of cats and aged ferrets as discussed below), which might be the major sticking barrier in the road to establish ideal SFTSV lethal models (Yu et al., 2011; Jin et al., 2012, 2015b; Liu Q. et al., 2014; Matsuno et al., 2017; Park E. S. et al., 2019). Thus, immunocompromised and newborn rodent models which are highly permissive for SFTSV infection and mimic some severe SFTS signs in humans, such as newborn mice, newborn rats, and IFN-signaling deficient mice, have been established to study the pathogenic mechanism and evaluate potential drugs and vaccines (Chen et al., 2012; Liu Y. et al., 2014; Gowen et al., 2017). In this paper, we review the advances and challenges in SFTSV animal model development by summarizing the characteristics of experimental animals infected with SFTSV and exemplifying some representative applications of the potential SFTSV animal models in studies of virus-host interactions, antiviral drugs, and vaccine candidates.

## Severe Fever With Thrombocytopenia Syndrome in Humans

The clinical manifestations of SFTS patients are extensive, ranging from mild fever to hemorrhagic fever, neurological presentations, multiple organ failure, and even death. Notably, acute fever, thrombocytopenia, leukocytopenia, bleeding tendency, and gastrointestinal symptoms are frequently observed in SFTS cases (Lei et al., 2015). The whole clinical disease course of the patients with SFTS encompasses four stages: incubation, fever, multiple organ failure, and convalescence (Liu S. et al., 2014). The incubation period lasts approximately 5 to 14 days since tick bites and before onset (Liu Q. et al., 2014). The second period, fever stage, ranges from 1 to 7 days, in which major manifestations are sudden attack of fever, headache, and gastrointestinal symptoms. Concurrently, the virus load reaches a high level in serum; blood tests show a progressive reduction of platelets (PLT) and white blood cells (WBC) and an increase of serum biochemical parameters including alanine aminotransferase (ALT), aspartate aminotransferase (AST), lactate dehydrogenase (LDH), blood urea nitrogen (BUN), and creatine phosphokinase (CPK) (Gai et al., 2012; Liu Q. et al., 2014; Liu S. et al., 2014; Li H. et al., 2018). After the fever stage, survivors have a convalescence, in which clinical signs begin to decrease and diminish, the blood plasma virus load drops gradually, and the abnormality of serum enzymes makes a recovery (Gai et al., 2012; Liu Q. et al., 2014; Liu S. et al., 2014). However, in severe cases, the disease may progress rapidly to the multiple organ failure stage that is closely related to the death. In this stage, the serum viral load of the patients remains high and serum biochemical parameters and hematological parameters remain markedly aberrant (Gai et al., 2012; Liu Q. et al., 2014; Li S. et al., 2018; Mendoza et al., 2019). Notably, patients who have the following features such as male sex, older age, high viral load, myalgia, hemorrhagic tendency, neurological signs, breathing difficulty, and some extremely abnormal laboratory variables, would have a higher risk of death (Gai et al., 2012; Li H. et al., 2018; He et al., 2020).

Several autopsy case reports showed that obvious pathological changes appeared in spleen, multiple lymph nodes, liver, bone marrow, kidney, gastrointestinal tract, heart, and lung (Hiraki et al., 2014; Takahashi et al., 2014, 2021; Uehara et al., 2016; Nakano et al., 2017; Kaneko et al., 2018; Saijo, 2018; Suzuki et al., 2020; Tsuru et al., 2021). Therein, spleen and multiple lymph nodes which were more severely infected in autopsy cases were more frequently observed with lesions. In an autopsy report of SFTS from Japan, severe necrotizing lymphadenitis with massive necrosis, depletion of small lymphocytes, and severe infiltration by histiocytes and immunoblasts were observed (Takahashi et al., 2014). Splenic lesions mainly including congestion, focal hemorrhage and ischemic lesions were observed in an aged male patient case (Li S. et al., 2018); there were necrocytosis with massive nuclear debris in white pulp and infiltration of numerous atypical large lymphocytes in red pulp and periarteriolar sheaths in a SFTS patient infected by bite of a cat carrying SFTSV (Tsuru et al., 2021). Lesions of liver principally involved multiple lobular necrosis, hemorrhage, and mild lymphocytic inflammation around the portal tracts, expansion of portal area, and acidophilic degeneration (Hiraki et al., 2014; Takahashi et al., 2014; Uehara et al., 2016; Li S. et al., 2018). In a subset of SFTS autopsy cases, the prominent hemophagocytosis also appeared in macrophages of lymph nodes, spleen, bone marrow and liver (Takahashi et al., 2014; Uehara et al., 2016; Nakano et al., 2017; Tsuru et al., 2021). A Chinese SFTS fatal case showed severe tubular distention and swollen renal tubular epithelial cells in kidney (Li S. et al., 2018). Moreover, several autopsy reports also documented lesions of other tissues such as neuronal degeneration, gastrointestinal bleeding, and structural disorders of myocardium cells (Kaneko et al., 2018; Li S. et al., 2018; Saijo, 2018). Additionally, there are reports showing lung damage in SFTS cases arising from severe secondary fungal infection (Hiraki et al., 2014; Uehara et al., 2016).

Recently, Suzuki et al. (2020) reported that B cells differentiating into plasmablasts and macrophages were targeted by SFTSV in lethal human infections and most of cell populations infected with SFTSV in multiple organs were B cell-lineage lymphocytes. Moreover, another study by Takahashi et al. (2021) demonstrated the infection of SFTSV in peripheral blood plasmablasts. These observations suggest that B cell-lineage lymphocytes are likely important targets of SFTSV in human infections, although more investigations are needed to fully understand the target cells of SFTSV infection and pathogenesis.

## Animal Models for Severe Fever With Thrombocytopenia Syndrome Virus

### Mice

**FIGURE 2:**
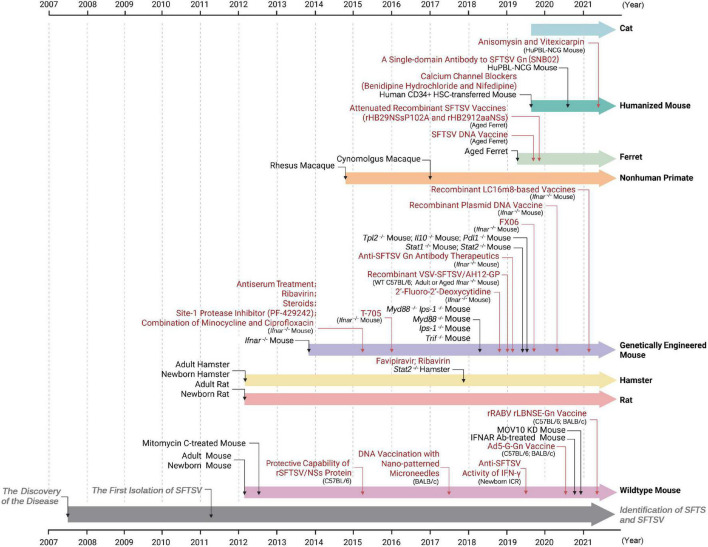
Timelines of events: development of SFTSV animal models and their applications in the studies of anti-SFTSV drugs and vaccines. The reporting time of SFTSV researches from 2007 (the year when SFTS cases were first recorded) to 2021 is shown. Animal models tested or applied at the indicated time are shown in black font. Application studies of the animal models at the indicated time are depicted in red font. Researches firstly reporting SFTS and isolating SFTSV virions are marked in gray. See text for details.

Mice have been a widely used model to study the viral pathogenic mechanisms, to understand key clinical features of SFTS, and to test the effectiveness of vaccine candidates and potential antiviral countermeasures following the isolation of SFTSV (as illustrated in Figure 2). As reported, researchers have attempted to use immunocompetent wildtype (WT) mice including C57BL/6 and BALB/c strains, suckling mice such as Kunming (KM), genetically modified or drug-treated mice with immune insufficiency, and some humanized mice with reconstituted immune systems (Figures 1, 2 and Table 1).

**TABLE 1:** Summary of the existing SFTSV animal models.

Animal model			SFTSV strain	Susceptibility to SFTSV	References
Mouse	Adult	C57BL/6, BALB/c, Kunming, ICR (CD-1), A/J, CAST/EiJ, DBA/1J, FVB/NJ, NZBWF1/J, BXD68/RwwJ, BXD34/TyJ, SJL/J, C3H, FVB, 129S1/svlmJ	Huaiyangshan-Human-1 of HYSV; HB29; YG-1; JS2011-013-1; SD4; CB1/2014	Non-lethal; hematologic abnormalities, aberrant regulation of proinflammatory cytokines and chemokines, dose-dependent effect; pathological lesions in liver and kidney	Chen et al., 2012, 2020; Jin et al., 2012; Liu et al., 2015; Sun et al., 2015; Matsuno et al., 2017; Park S. J. et al., 2019; Park et al., 2020
	Newborn	C57BL/6, BALB/c, Kunming, ICR (CD-1)	Huaiyangshan-Human-1 of HYSV; YL-1	Lethal; clinical symptoms (Kunming), neurological signs (Kunming); pathological lesions in liver and neuron (Kunming)	Chen et al., 2012; Liu Y. et al., 2014; Ning et al., 2019
	Mitomycin C-treated	C57BL/6	HB29	Lethal (50% mortality)	Jin et al., 2012
	IFNAR Ab-treated	C57BL/6	KH1	Lethal; weight loss, hematologic abnormalities; pathological lesions in spleen, liver and intestinal tract	Park et al., 2020
	*Ifnar* ^–/–^	C57BL/6, 129/Sv	YG-1; SPL010; SD4; HB29; YL-1; KH1; USAMRIID-HLP23	Lethal (100% mortality); vascular leak; pathological lesions	Liu Y. et al., 2014; Tani et al., 2016; Matsuno et al., 2017; Choi et al., 2019; Westover et al., 2019; Yoshikawa et al., 2019; Kang et al., 2020; Park et al., 2020; Perez-Sautu et al., 2020
	Aged	C57BL/6, BALB/c, 129S1/svlmJ	CB1/2014; SD4	Non-lethal; moderate weight loss	Matsuno et al., 2017; Park S. J. et al., 2019
	*Stat1* ^–/–^	C57BL/6	YG-1	Non-lethal; mild symptoms	Yoshikawa et al., 2019
	*Stat2* ^–/–^	C57BL/6	YG-1	Lethal (100% mortality)	Yoshikawa et al., 2019
	Pregnancy	C57BL/6	JS2011-013-1	Non-lethal (maternal mouse); placental damage, fetal reabsorption and IUGR; vertical transmission	Chen et al., 2020
	*Ips-1* ^–/–^	C57BL/6	SPL010	Non-lethal; viremia	Yamada et al., 2018
	*Myd88* ^–/–^	C57BL/6	SPL010	Lethal (20% mortality); viremia	Yamada et al., 2018
	*Ips-1*^–/–^ *Myd88*^–/–^	C57BL/6	SPL010	Non-lethal	Yamada et al., 2018
	MOV10 KD	C57BL/6	WCH	Non-lethal; hematologic abnormalities	Mo et al., 2020
	Humanized	Human CD34 + HSC-transferred mouse, human PBMCs-transferred mouse (HuPBL–NCG mouse)	WCH; E-JS-2013-24	Lethal; hematologic abnormalities, weight loss, vascular leak; pathological lesions in spleen, liver, lung and kidney;	Li et al., 2019; Wu et al., 2020; Xu et al., 2021
Rat	Adult	Wistar rat	Huaiyangshan-Human-1 of HYSV	Non-lethal	Chen et al., 2012
	Newborn	Wistar rat	Huaiyangshan-Human-1 of HYSV	Lethal	Chen et al., 2012
Non-human primate	Adult	Rhesus macaque, Cynomolgus macaque	HB29; SD4	Non-lethal; no or mild symptoms; pathological lesions in liver and kidney	Jin et al., 2015b; Matsuno et al., 2017
Hamster	Adult	Syrian golden hamster	Huaiyangshan-Human-1 of HYSV; HB29; YL-1	Non-lethal; pathological lesions in liver and kidney	Chen et al., 2012; Jin et al., 2012; Liu Y. et al., 2014
	Newborn	Syrian golden hamster	Huaiyangshan-Human-1 of HYSV; YL-1	Non-lethal; asymptomatic	Chen et al., 2012; Liu Y. et al., 2014
	*Stat2* ^–/–^	Syrian golden hamster	HB29	Lethal; hematologic abnormalities; pathological lesions in liver and spleen	Gowen et al., 2017
Ferret	Adult (≤2 years old)	*Mustela putorius furo*	CB1/2014	Non-lethal; Asymptomatic	Kwak et al., 2019; Park S. J. et al., 2019; Yu et al., 2019a,b; Yun et al., 2020
	Aged (≥4 years old)	*Mustela putorius furo*	CB1/2014	Lethal (93% mortality); hematologic abnormalities, age-dependent effect	Kwak et al., 2019; Park S. J. et al., 2019; Yu et al., 2019a,b; Yun et al., 2020
Cat	Adult (2 years old); Newborn (0.5 year old)	Russian Blue, American Shorthair	SPL010	Lethal (4 of 6 cats); fever, weight loss, hematologic abnormalities; pathological lesions in the lymph nodes, spleen and gastrointestinal tract	Park E. S. et al., 2019

#### Immunocompetent Adult Mice

Jin et al. (2012) first described the characteristics of immunocompetent mouse infected with SFTSV in adult C57BL/6 strain in detail. The C57BL/6 mice incubated with SFTSV (HB29 strain, 10^5^ TCID_50_) by intramuscular (i.m.) route developed some characteristic abnormalities of SFTS in a short period of time post-infection (p.i.), having onset of leukocytopenia at day 1 p.i. and thrombocytopenia at day 3 p.i., showing an increase of ALT, AST and BUN, and shedding SFTSV via various excreta. All survived and clinical symptoms such as fever and body weight loss were not observed (Jin et al., 2012; Park et al., 2020). Viral replication was only detected in the spleen, indicating that spleen might be the target organ of SFTSV in the model (Jin et al., 2012). Transient pathological lesions were self-recovering within 28 days p.i. In the early stage of infection, the pathological lesions were mainly in spleen and bone marrow, showing that megakaryocytes (the main hematopoietic cell) increased in both spleen and bone marrow, and lymphocytes of the red pulp decreased in spleen (Jin et al., 2012). In the late stage of infection, the changes were noted in liver and kidney, mainly manifesting acute glomerular nephritis and acute hepatitis with self-limiting outcomes (Jin et al., 2012).

Overall, the adult C57BL/6 model probably mimics parts of characteristics of human infections, but has significant limitation that it cannot develop into a severe disease or die of the infection. Similarly, other immunocompetent adult mouse strains infected with SFTSV, including BALB/c, C3H, FVB, and ICR (CD-1), also lack severe clinical manifestations or mortality (Jin et al., 2012, 2015a; Matsuno et al., 2017; Park S. J. et al., 2019; Table 1).

The adult wildtype mice have been used in researches on targeting of SFTSV infection, evaluation of safety and efficacy of candidate vaccines and therapeutics, and transmission modes of SFTSV (Figure 2). For instance, by using the C57BL/6 mouse model, researchers found that SFTSV stimulated production and secretion of pro-inflammatory cytokine Interleukin (IL)-1β and further confirmed that the NLRP3 (NLR family, pyrin domain containing 3) inflammasome complex acted as an essential mediator in the process (Liu et al., 2021). Several vaccine candidates based on viral vectors, expression plasmids, or the viral protein (NSs) have been evaluated in wildtype C57BL/6 or BALB/c mice (Jung et al., 2017; Zhao et al., 2020; Tian et al., 2021). Therein, immunization with recombinant SFTSV NSs protein could induce a high titer of anti-NSs antibody in sera and yet it could not promote SFTSV clearance in mice (Liu et al., 2015). Two recombinant viral vectors including a recombinant rabies virus (RABV) expressing SFTSV Gn (rLBNSE-Gn) and a recombinant human adenovirus type 5 co-expressing RABV G and SFTSV Gn (Ad5-G-Gn) were designed as potential bivalent vaccines against SFTSV and RABV to take precaution against possibly dangerous contacts and infection between animals and humans (Park E. S. et al., 2019; Yamanaka et al., 2020; Zhao et al., 2020; Ando et al., 2021; Tian et al., 2021). Additionally, a recent study showed that SFTSV can be transmitted vertically across the placental barrier of the pregnant maternal mice (C57BL/6) to the fetuses, resulting in widely distributed infection in multiple organs of the fetuses (Chen et al., 2020). Thus, the immunocompetent C57BL/6 mouse model also might serve for investigating maternal transmission of SFTSV.

#### Immunocompromised Adult Mice

To overcome the host restriction of SFTSV pathogenicity, immunocompromised adult mice including IFN-α/β receptor knockout (*Ifnar*^–/–^) or STAT2 knockout (*Stat2*^–/–^) mice and those treated with blocking antibody against IFNAR (IFNAR Ab) or mitomycin C have been used for SFTSV infection. As expected, these animals were indeed demonstrated to be relatively susceptible to SFTSV challenge and displayed some severe clinical manifestations of human infection (Figure 1 and Table 1; Chen et al., 2012; Jin et al., 2012; Liu Y. et al., 2014; Matsuno et al., 2017; Westover et al., 2019; Yoshikawa et al., 2019; Park et al., 2020; Perez-Sautu et al., 2020).

##### Mitomycin C-Treated Mice

Mitomycin C-treated mice were the first immunocompromised model devoted to SFTSV researches (Jin et al., 2012). Mitomycin C is an inhibitor of hematopoiesis of bone marrow. C57BL/6 mice treated with mitomycin C (0.02 mg per mouse) via intraperitoneal (i.p.) route daily from 3 days before to 3 days after challenge with SFTSV (10^5^ TCID_50_, i.m.) developed more severe symptoms than the control group (Jin et al., 2012). Half succumbed within 10 dpi and surviving mice were identified to lose weight (Jin et al., 2012). Additionally, following challenge with SFTSV, pregnant mice (C57BL/6) treated with mitomycin C seemed to have severer placental necrosis and resorption of fetus, compared to the control group without the drug treatment (Chen et al., 2020). Taken together, the mitomycin C-treated mouse model might be more susceptible to SFTSV, compared to the untreated mice; however, the studies about the model are still scarce and it remains to be further determined whether or in what cases it is suitable as a SFTSV lethal model. Particularly, the mechanism underlying the permissiveness of the model to SFTSV needs to be clarified.

##### Ifnar^–/–^ Mice

*Ifnar*^–/–^ mice, as a common model for *in vivo* studies of many viral infections, have been given much attention in SFTSV studies. *Ifnar*^–/–^ mice can develop lethal signs and replicate severe disease manifestations of humans even with low dosage of SFTSV challenge (Liu Y. et al., 2014; Tani et al., 2016; Matsuno et al., 2017; Westover et al., 2019; Yoshikawa et al., 2019; Park et al., 2020). Originally, a study by Liu Y. et al. (2014) reported that *Ifnar*^–/–^ mice were highly susceptible to SFTSV (YL-1) and the virus replicated abundantly in multiple organs of the model, quickly reaching high levels. While the animals died a few days after SFTSV infection, no significant histopathological lesions were observed by the authors (Liu Y. et al., 2014). Later, other researchers further successively described the clinical signs in SFTSV-infected *Ifnar*^–/–^ mice that developed fatal illness. In these following studies, *Ifnar*^–/–^ mice infected with SFTSV exhibited remarkable hematologic changes like severe viremia and leukocytopenia, developed clinical manifestations such as severe weight loss, ruffled fur, and depression, and succumbed to the infection (Tani et al., 2016; Matsuno et al., 2017; Westover et al., 2019; Park et al., 2020). Significant histopathological abnormalities of spleen, liver, kidney, lymph nodes, and bone marrow were common, among which spleen was likely the major target organ with the highest virus titers (Tani et al., 2016; Matsuno et al., 2017; Westover et al., 2019; Park et al., 2020). Lesions of spleen predominantly consisted of histiocytic and necrotizing splenitis, white pulp atrophy, and diffuse reticuloendothelial hyperplasia of red pulp (Tani et al., 2016; Matsuno et al., 2017; Park et al., 2020). In addition, similar to human severe cases, Westover et al. (2019) reported SFTSV-induced vascular fibrinoid necrosis in spleen in *Ifnar*^–/–^ mice, which was accompanied by increased pro-inflammatory cytokines such as IL-6, monocyte chemoattractant protein-1 (MCP-1), tumor necrosis factor (TNFα), IFN-γ, RANTES, and IL-1β in serum, spleen and other tissues. There were pyknosis and karyorrhexis of lymphocytes in both spleen and cervical lymph nodes and histiocytic and necrotizing lymphadenitis lesions in cervical lymph nodes at the late stage of infection (Matsuno et al., 2017). The liver lesions included acute, multifocal necrotizing neutrophilic and histiocytic hepatitis, while acute tubular epithelial necrosis at the corticomedullary junction and deep cortex were observed in kidney (Tani et al., 2016; Westover et al., 2019; Park et al., 2020). Moreover, there were minimal to moderate necrosis with edema and fibrin in bone marrow, and prominent damage of small intestinal villi observed in this model (Matsuno et al., 2017; Park et al., 2020).

Using the *Ifnar*^–/–^ mouse model, broad-spectrum antiviral drugs including T-705 (Favipiravir) (Tani et al., 2016, 2018), 2′-Fluoro-2′-Deoxycytidine (Smee et al., 2018), and ribavirin (Shimada et al., 2015; Tani et al., 2016) have been shown to reduce the mortality rate and viral titers of SFTSV *in vivo*, albeit with varying efficiency (Takayama-Ito and Saijo, 2020). Further, after vaccination, complete protection for *Ifnar*^–/–^ mice upon lethal challenge of SFTSV was provided by a recombinant plasmid DNA co-expressing SFTSV antigens (Gn, Gc, NP, and NSs) and IL-12 (Kang et al., 2020) and two SFTSV Gn/Gc-encoding recombinant viral vectors respectively based on attenuated vaccinia virus (Yoshikawa et al., 2021) or vesicular stomatitis virus (Dong et al., 2019). In addition, antiserum treatment, anti-SFTSV Gn antibody therapeutics, and a combination medication of minocycline (MINO) and ciprofloxacin (CPFX) also had encouraging consequences in treatment of SFTSV infection (Shimada et al., 2015; Kim et al., 2019). Whereas, administration of FX06, a natural plasmin digest product of fibrin clinically used as a treatment for vascular leak, was confirmed to restrain SFTSV-induced vascular leak to certain extent but fail to significantly increase survival rate of *Ifnar*^–/–^ mice (Westover et al., 2019).

With high susceptibility to SFTSV and severe attacking characteristics, the *Ifnar*^–/–^ mice have become the most widely used SFTSV animal model for exploring the pathogenesis of acute fatal disease course and for evaluating safety and efficacy of candidate vaccines and therapeutics (as shown in Figure 2). At the same time, it should also be acknowledged that the *Ifnar*^–/–^ mouse model has shortcomings that cannot be ignored in many studies. Particularly, due to lack of the initial antiviral response mediated by type I IFNs, the animals cannot effectually replicate the interactions of the virus infection with the innate immune system. Moreover, *Ifnar*^–/–^ mice have quick onset of disease and generally succumb from an acute disease course, resulting in incomplete delineation of the full course of SFTS and great difficulty in fully recapitulating features of human cases.

##### IFNAR Ab-Treated Mice

An IFNAR Ab-treated mouse model was established by i.p. injection of C57BL/6 mice with reversibly blocking monoclonal antibody against IFNAR (MAR15A3) 1 day before and 2 days after SFTSV infection (Park et al., 2020; Figure 2 and Table 1). The susceptibility of the IFNAR Ab mice was then compared with those of untreated WT or *Ifnar*^–/–^ mice by challenge of SFTSV (KH1) in doses of 5 × 10^2^ to 5 × 10^5^ FAID_50_ via i.p. injection (Park et al., 2020). Consistent with the previous studies, the WT mice survived all SFTSV infective doses, while the *Ifnar*^–/–^ mice all died after infection even with the lowest dose of SFTSV within 4 days p.i. (Park et al., 2020). Interestingly, although SFTSV infection with doses of 5 × 10^3^ to 5 × 10^5^ FAID_50_ also resulted in death of the IFNAR Ab mice, the survival time appeared to be prolonged in comparison with the *Ifnar*^–/–^ group (Park et al., 2020). Moreover, in the dose of 5 × 10^2^ FAID_50_, the IFNAR Ab mice did not succumb to SFTSV infection and recovered their lost weight after 7 days p.i. (Park et al., 2020). Further, the RNA levels in tissues and excreta and pathological lesions were also compared in the three mouse groups infected with SFTSV of 5 × 10^2^ FAID_50_. Although the viral RNA levels in organs were initially detected with no significant difference in the control, IFNAR Ab, and *Ifnar*^–/–^ mice, after 3 dpi the *Ifnar*^–/–^ mice exhibited significantly more SFTSV RNA copies than the other two groups (Park et al., 2020). Additionally, viral RNA in the IFNAR Ab and *Ifnar*^–/–^ mice appeared earlier than that of the WT control mice in the eye and oral swabs and urine, and compared to the control mice, the IFNAR Ab group showed longer viral shedding (up to 7 days p.i.) (Park et al., 2020). Histopathologically, the IFNAR Ab mice with SFTSV infection developed significant but self-restorative lesions including coagulation necrosis and mononuclear inflammatory infiltration in liver, white pulp atrophy of spleen, and injury of intestinal villi at day 7 p.i. However, these lesions seemed milder than those in the *Ifnar*^–/–^ mice (Park et al., 2020). Together, the sensitivity of IFNAR Ab mice appears to be intermediate between WT and *Ifnar*^–/–^ mice and these IFNAR Ab-treated animals might be used as an alternative model to make up for the deficiency of *Ifnar*^–/–^ mouse.

##### Stat2^–/–^ and Stat1^–/–^ Mice

STAT1 and STAT2 are key components of type I and III IFN signaling pathways (Darnell et al., 1994; McNab et al., 2015). Moreover, a previous study in our lab has established that the NSs protein of SFTSV sequestrates human STAT2 and STAT1 into viral inclusion bodies and hence efficiently antagonizes the antiviral IFN signaling cascades in human cells (Ning et al., 2015). In view of these, several *Stat1*^–/–^ or *Stat2*^–/–^ animal models have been tested for the susceptibility to SFTSV infection (Gowen et al., 2017; Yoshikawa et al., 2019; Figures 1, 2 and Table 1). In a study by Yoshikawa et al., the infectivity and pathogenicity of SFTSV in *Stat1*^–/–^, *Stat2*^–/–^, *Ifnar*^–/–^, or WT mice were compared (Yoshikawa et al., 2019). As reported, *Stat2*^–/–^ and *Ifnar*^–/–^ mice all sacrificed 4–8 days p.i. with weight loss, leukocytopenia, and thrombocytopenia in SFTSV (YG-1) lethal challenge, and variation trends and amplitudes of parameters between the two groups were highly consistent (Yoshikawa et al., 2019). However, *Stat1*^–/–^ mice all survived only with transient symptoms and WT mice were asymptomatic (Yoshikawa et al., 2019). Consistently, the viral titers detected in organs of *Stat2*^–/–^ mice were as high as those in *Ifnar*^–/–^ mice but much higher than those in *Stat1*^–/–^ mice, suggesting the possibly more important roles of STAT2 or the antiviral genes specifically induced by STAT2 in restriction of SFTSV replication (Yoshikawa et al., 2019). In addition, the participation of STAT2 occurs exclusively in the type I and III IFN antiviral responses, whereas STAT1 is instrumentalized in many signaling pathways directed by not only the antiviral IFNs but also inflammatory cytokines such as IFN-γ, IL-6, and IL-17 (Darnell et al., 1994; Wegenka et al., 1994; Subramaniam et al., 1999; Hoffmann et al., 2015; Ning et al., 2019). Thus, *Stat1* knockout might simultaneously lead to the attenuation of excessive inflammatory reactions and the resultant lesions, perhaps decreasing the disease severity, although further detailed experimental analyses are needed to fully understand the differential susceptibility of *Stat1*^–/–^ and *Stat2*^–/–^ mice to SFTSV infection. Subsequently, as hypothesized in the previous study (Ning et al., 2015), Yoshikawa et al. (2019) demonstrated that in contrast to the human counterpart, murine STAT2, indeed, cannot be targeted by SFTSV NSs, resulting in the inability of NSs to suppress antiviral IFN signaling in cultured murine cell lines. It may at least partially explain the species-specific pathogenicity of SFTSV (in mice vs. humans). Future studies *in vivo*, e.g., those with STAT2 humanized mice, not only would further corroborate the *in vitro* findings but also may help inform the development of an immunocompetent and probably better animal model for SFTSV infection. Together, the *Stat2*^–/–^ mice can develop severe SFTS-like disease and could be used as a lethal model for preliminary efficacy testing of intervention strategies combating SFTSV, while the *Stat1*^–/–^ mice might be a candidate of non-lethal models, although further delineations of these gene-deficient animals infected with SFTSV are merited, especially in histopathology.

#### Age-Dependent Mouse Models (Newborn and Aged Mice)

The susceptibility of mice with different ages to SFTSV has been tested to elucidate whether the sensitivity of animals to the virus depends on age, to find the age-specific characteristics, and finally to reveal the pathogenesis of diseases at different age stages (Table 1). As reported, newborn mice with various backgrounds of strains (such as KM, C57BL/6, BALB/c, and ICR) are sensitive to SFTSV and develop severe symptoms and high mortality (Chen et al., 2012; Liu Y. et al., 2014; Ning et al., 2019). Newborn KM, BALB/c, and C57/BL6 mice (1–3 days old) with intracerebral (i.c.) infection in a dose of 2 × 10^7^ copies all died, while 35–50% of those with i.p. infection in a dose of 3 × 10^7^ copies sacrificed (Chen et al., 2012). Newborn KM mice intraperitoneally infected with SFTSV (6 × 10^5^ to 6 × 10^8^ copies per animal) had signs of ruffled fur, weight loss, superexcitation, rear limb weakness or paralysis (Chen et al., 2012). Histopathological analysis of the KM mice dying from 6 × 10^5^ i.p. infection displayed that there was soakage of considerable mononuclear cells and obvious large necrotic areas in livers, infiltration of inflammatory cells and necrotic areas in neurons, but no lesions in lung, heart, spleen, or kidney (Chen et al., 2012). ICR suckling mice (3 days old) were also shown to be highly susceptible and the challenge of 1.5 × 10^3^ TCID_50_ by i.c. rendered their 100% mortality within 13 days p.i. (Ning et al., 2019). On the basis, the anti-SFTSV activity of IFN-γ was evaluated in the newborn ICR mouse model, confirming that treatment with IFN-γ prior to the viral infection has inhibitory activity against SFTSV *in vivo* (Ning et al., 2019).

Although the newborn mice are highly susceptible to SFTSV and have pathological lesions when responding to SFTSV infection, their limitations are obvious. Firstly, the differences between immune systems of newborns and adults are quite large. For instance, newborn mice have an immature immune system to respond viral antigens, as lymphoid follicles, germinal center structures of secondary lymphoid tissue and antigen-presenting cells considered to be essential for adaptive immunity are not fully developed in days-old neonates (Fu and Chaplin, 1999; Adkins et al., 2004; Basha et al., 2014). In addition, some experiments on suckling mice are difficult to operate (e.g., collection of blood samples is hard to achieve). Thus far, research on newborn mice is still partial, and the pathological features needs to be further characterized.

In contrast to the newborns, aged mice of 129S1/svlmJ and C57BL/6J strains (12–24 months old) inoculated with SFTSV at a high dose of 10^5^ TCID_50_ or a low dose of 10^2^ TCID_50_ via intradermal (i.d.), subcutaneous (s.c.), i.m. or i.p. routes all survived the experimental infections, and did not develop any evident pathological lesions, except that the aged C57BL/6 mice infected with SFTSV via s.c. route had symptoms of weight loss (Matsuno et al., 2017). In another study, aged mice (BALB/c, C3H, C57BL/6, and FVB strains, ≥20 months old) infected with SFTSV (10^7.6^ TCID_50_) also merely exhibited a slight weight loss and recovered a few days later (Park S. J. et al., 2019). Collectively, these results demonstrate that the aged mice are not more susceptible to SFTSV than the adults or newborns. Synthesizing the results drawn from the newborn, adult, and aged mice, unlike humans, aging seems not a main risk factor of SFTS progression for mice, and the age-associated mouse models are likely inappropriate to simulate the characteristics of the worsened SFTS in elderly human patients or to study the age-specific pathogenesis of SFTSV.

#### Humanized Mouse Models

In last few years, two kinds of humanized mice constructed by immune system reconstitution have been reported as SFTSV lethal models, which could mimic some important aspects in human infection more appropriately than wild type and immunocompromised mice (Li et al., 2019; Wu et al., 2020; Xu et al., 2021; Figure 2). It opens new doors for SFTSV mouse modeling that mice transplanted with human CD34 antigen-positive (CD34+) hematopoietic stem cells (HSCs) or with human peripheral blood mononuclear cells (PBMCs) have been successively attempted in SFTSV research.

Humanized mouse models offer obvious advantages. Key clinical features that the humanized mice share with humans include systemic infection, abnormalities of serum enzymes, thrombocytopenia and leukocytopenia (Li et al., 2019; Xu et al., 2021). Further, obvious pathological lesions of sacrificed humanized mice with engraftment of human PBMCs (HuPBL-NCG mice) involve megakaryocyte infiltration and lymphocyte depletion in the red pulp of spleen, obvious peribronchiolar inflammation in lung, and hepatocytic degeneration and scattered necrosis in liver, which also is coincident with multiple organ failure in fatal human cases (Xu et al., 2021). In addition, it is of interest that severe vascular leak regarded as a higher risk of death in human cases was observed in the human PBMC-transferred mice challenged with SFTSV. Moreover, differing from the refractory wild type and the extremely vulnerable immunocompromised mice, the humanized mice showed moderate susceptibility to SFTSV. They had longer time to struggle with the infection and some of them survived (Li et al., 2019; Wu et al., 2020; Xu et al., 2021), which provides the extended observation window for research on the pathogenesis of SFTS and evaluation of vaccines and drug therapies. Additionally, the models possess the advantages of simple operation and quick acquisition.

Until now, several drug or antibody administrations have been confirmed to significantly decrease mortality rates of SFTS in the human CD34 + HSC-transferred mice (two calcium channel blockers, benidipine hydrochloride and nifedipine) and the HuPBL-NCG mice (two candidate drugs from an *in vitro* screen, anisomycin and vitexicarpin, and a single-domain antibody to SFTSV Gn, SNB02) (Li et al., 2019; Wu et al., 2020; Xu et al., 2021; Figure 2). In addition, the humanized mouse models also may have the value of exploring the SFTSV pathogenic mechanisms. In SFTSV-infected HuPBL-NCG mice, Xu et al. (2021) found that the continuity and integrity of endothelial cells were disrupted and vascular permeability was increased. Furthermore, the authors suggested that apoptosis and VE-cadherin internalization of endothelial cells and the release of cytokines induced by SFTSV infection might promote vascular injury in the HuPBL-NCG mouse model (Xu et al., 2021). The findings highlight the potential applicability of the model in the study of virus-induced hemorrhage syndrome.

A number of limitations of the humanized mouse models also should be acknowledged. Immune rejection reaction likely occurs in some humanized mice like the HuPBL-NCG, leading to graft-vs.-host disease (GVHD) that could interfere with the observation of SFTSV-induced abnormality and result in inaccuracy, confusion, and inconsistency. The limited lifespan of these models may hamper adequate evaluation of some vaccination strategies (Zitvogel et al., 2016; Walsh et al., 2017; Allen et al., 2019). In addition, due to individual differences of donors, the degree of immune system reconstruction could be inhomogeneous in mice. Thus, these humanized models may have significant variations and weak stability.

#### Other Mouse Models

Bone marrow (BM)–chimeric mice with IFNAR1, IFN-β promoter stimulator 1 (IPS-1) or myeloid differentiation factor 88 (MyD88) deficient hematopoietic cells were generated in a study (Yamada et al., 2018). IPS-1 (also known as VISA, MAVS, or CARDIF) and MyD88 are essential signaling adaptors for the IFN and inflammatory cytokine induction pathways mediated by retinoic acid-inducible gene I (RIG-I)-like receptors (RLRs) and Toll-like receptors (TLRs), respectively (Takeda and Akira, 2004; Kawai et al., 2005; Meylan et al., 2005; Seth et al., 2005; Xu et al., 2005). Using these BM-chimeric mouse models, the authors suggested that excessive induction of inflammatory cytokines might be a key cause of lethal SFTS, despite the important anti-SFTSV role of IFN-I signaling (Yamada et al., 2018). In addition, a study using mouse models with tumor progression locus 2 (TPL2) or IL-10 knockout together with IFN signaling deficiency revealed that IL-10 induced by TPL2 signaling pathway might lessen host immune response and enhance SFTSV infection (Choi et al., 2019). In our previous study, the host protein moloney leukemia virus 10 (MOV10) was demonstrated to directly prevent the viral RNP assembly by targeting SFTSV N protein, interfering with virus replication and propagation (Mo et al., 2020). Moreover, MOV10-knockdown C57BL/6 mouse conducted by lentiviral vectors expressing specific shRNA was designed to further demonstrate the anti-SFTSV activity of MOV10. In SFTSV challenge, MOV10 knockdown resulted in increased viral loads in organs and serum as well as lower platelet count and elevated ALT level, corroborating the role of MOV10 in restricting SFTSV infection and pathogenicity (Mo et al., 2020). Although no severe clinical manifestations or death were observed in the MOV10-knockdown mice, the study highlights the potential significance of decipherment of host restriction factors or pro-viral factors through the study of virus-host interactions for development of specific SFTSV animal models.

### Hamsters

Aside from mouse models, Syrian golden hamster (*Mesocricetus auratus*) was another informative rodent pressed into service in studies of SFTSV infection and evaluation of the curative effect of antiviral drugs (Figures 1, 2 and Table 1). Until now, newborn, adult, and STAT2-deficient hamsters have been used in SFTSV infection experiments (Chen et al., 2012; Jin et al., 2012; Liu Y. et al., 2014; Gowen et al., 2017). Of concern is a significant difference from mice that newborn hamsters inoculated with SFTSV do not exhibit more severe illness compared with adults, which is probably attributed to differences in species between hamster and mouse (Chen et al., 2012; Jin et al., 2012; Liu Y. et al., 2014). Like other SFTSV-infected adult rodents, adult hamsters merely developed slight pathological changes in liver and kidney without obvious clinical manifestations (Jin et al., 2012). Further, STAT2-deficient hamsters were tested and established as a lethal model to validate the significance of STAT2 in control of SFTSV infection (Gowen et al., 2017). The *Stat2*^–/–^ hamster model was highly susceptible to SFTSV and showed the characteristics of SFTSV infection in humans including viremia, thrombocytopenia, weight loss, and severe pathological lesions accompanied by neutrophilic inflammation in liver and white pulp of spleen; however, in contrast to human infections, no significant change in white blood cell count was observed (Gowen et al., 2017). Besides, the biochemistry parameters of renal function had an increase without histopathological lesions in kidney microscopically (Gowen et al., 2017). In addition to analyzing the role of STAT2 in resisting SFTSV infection, the model is an option to study potential therapeutic treatments against SFTSV infection. However, the differences in SFTSV-infected hamsters with human patients and other models could not be overlooked. Notably, favipiravir has been confirmed to provide a complete protection against the lethal SFTSV infection for *Stat2*^–/–^ hamsters in accord with the conclusion drawn from *Ifnar*^–/–^ mice, while ribavirin that provides protection for *Ifnar*^–/–^ mice is ineffective for *Stat2*^–/–^ hamsters against SFTSV (Shimada et al., 2015; Tani et al., 2016, 2018; Yoshikawa et al., 2019).

### Rats

Compared with other laboratory rodents, rats are rarely seen as a model of SFTSV infection although newborn rats also develop lethal SFTS disease resembling newborn mice. In the research by Chen et al., newborn Wistar rats (1–3 days old) challenged with SFTSV via i.c. route in a dose of 2 × 10^7^ copies all died, while 40% of those infected via i.p. route in a dose of 3 × 10^7^ copies sacrificed (Chen et al., 2012). By contrast, all adults infected with SFTSV (3 × 10^7^ copies by i.c. route or 1.25 × 10^8^ copies by i.p. route) survived and had no obvious symptoms during 25-day observation (Chen et al., 2012). The characteristics of SFTSV rat models have yet to be supplemented and in particular, histopathological and hematological examinations have not been reported so far.

### Ferrets

Considering the similar anatomical and physiological features with human, ferrets have become a valuable experimental animal serving in the research fields of life sciences such as virology and immunology (Imai et al., 2012; Albrecht et al., 2018; Kim et al., 2020). Recently, Park et al. analyzed the susceptibility of outbred ferret models (*Mustela putorius furo*) to SFTSV (CB1/2014), establishing young adult (≤2 years of age) and aged (≥4 years of age) ferret models on the basis of the age-dependent characteristic of SFTS in human (Zhang et al., 2013; Park S. J. et al., 2019; Figures 1, 2 and Table 1).

The young and aged ferrets both had the same treatment with SFTSV at a dosage of 10^7.6^ TCID_50_ by i.m. route (Park S. J. et al., 2019). The ferrets in young adult group were semi-susceptible to SFTSV and all survived (Park S. J. et al., 2019). They merely displayed mild symptoms, such as 4–5% loss in mean weight and slightly rising body temperature, and recovered during 16-day observation (Park S. J. et al., 2019). Hematological analysis showed that PLT and WBC were kept in the normal range with a transient and slight drop (Park S. J. et al., 2019). AST and ALT had similar variation tendency with a noticeable rise and reached the peak at 4 dpi, but declined gradually within normal limits (Park S. J. et al., 2019). The viral RNA was detectable in spleens, livers, kidneys, lungs, and sera of the young ferrets (Park S. J. et al., 2019). In comparison with the young group, aged ferrets simulated the abnormalities of the lethal cases in humans more accurately (Park S. J. et al., 2019). The aged ferrets exhibited a higher mortality rate (up to 93%) and more severe clinical symptoms, such as fever, rapid loss of body weight, more rapid and severe thrombocytopenia and leukopenia, and continuous increase of ALT and AST in serum (Park S. J. et al., 2019). WBC and PLT count had a sustained decline, beginning at 2 dpi and gradually dropping below the normal range until death at 6–8 dpi (Park S. J. et al., 2019). Park S. J. et al. (2019) also detected viral RNA in serum, brain, lung, liver, spleen, kidney, intestine, and spinal cord tissues of the infected aged ferrets, demonstrating that systemic infection also occurs in the aged group. Therein, spleen was considered the most vulnerable organ since it had the highest viral load in both young adult and aged ferrets (Park S. J. et al., 2019). Histopathologically, pathological lesions were demonstrated more severe in aged ferrets (Park S. J. et al., 2019). Moreover, in follow-up examination of the susceptibility of ferrets to other SFTSV isolates, young ferrets survived infections of all SFTSV isolates and had no weight loss, while significant differences in pathogenicity among various SFTSV strains were observed in aged ferrets, indicating that the virulence of different SFTSV genotypes are diverse (Yu et al., 2019b; Yun et al., 2020). Taken together, the young ferrets might be used as a model to recapitulate mild SFTS cases of humans; the aged ferrets are more susceptible and can be a lethal model for SFTSV, while the virus strain employed should be also carefully chosen (Park S. J. et al., 2019; Yu et al., 2019a).

Compared with lethal models of rodents, immunocompetence makes the aged ferret model possess certain advantages. As reported, SFTSV infection leads to activation and dysregulation of key immune and inflammatory responses involving IFN signaling, IL-6 signaling, dendritic cell maturation, and leukocyte extravasation in aged ferrets, implying that the aged ferret model might be effectively devoted to test the correlation of immune and inflammatory signaling with SFTSV infection and pathogenicity (Park S. J. et al., 2019). Additionally, the aged ferret model may have predictive value of vaccine efficacy. In total, one DNA vaccine candidate based on plasmids expressing the viral proteins and two live-attenuated virus vaccine candidates with NSs mutations have been evaluated in the aged ferret model and both were demonstrated to induce neutralizing antibody and provide sufficient protection against lethal SFTSV (CB1/2014 strain) challenge (Kwak et al., 2019; Yu et al., 2019b; Figure 2). Besides, the aged ferret model also has been applied for research of SFTSV shedding and transmission mode, as it was reported that large batches of SFTSV might be excreted through nasal discharge, saliva and urine lasting for more than 12 days (Yu et al., 2019a). Despite its probable advantages in the SFTSV infection studies, some limitations still hamper continued applications of the model. For instance, inbred, specific-pathogen-free (SPF) and aged ferrets are scarce. Moreover, the experiments performed with ferrets are also limited by high cost, complexity of experimental operations, and paucity of commercial immunoreagents.

### Non-human Primates

To date, two studies that investigated SFTSV infection in rhesus macaques (Jin et al., 2015b) and cynomolgus macaques (Matsuno et al., 2017), respectively, have been reported, both indicating that SFTSV would not cause a fatal infection in macaques (Figures 1, 2 and Table 1). In all four cynomolgus macaques infected with SFTSV (SD4) in a dose of 10^6^ TCID_50_ by s.c. route, there were no visible clinical signs, no detectable viruses in blood, and no histopathological changes observed during the experimental period (Matsuno et al., 2017). Only one of the four cynomolgus macaques had a transient decrease in platelet amount (Matsuno et al., 2017). In comparison, rhesus macaques injected with SFTSV (HB29) in a dose of 10^7^ TCID_50_ via i.m. route had the typical manifestations of fever, thrombocytopenia, and leukocytopenia, although they recovered without any management, suggesting that rhesus macaques could develop mild symptoms and might be more susceptible to SFTSV than cynomolgus macaques (Jin et al., 2015b). Furthermore, the SFTSV-infected rhesus macaques showed some histopathological lesions including multifocal mild-to-moderate piecemeal necrosis and bridging necrosis of hepatocytes, mild hepatocyte degeneration, and inflammatory cell infiltration in the liver and glomerular hypercellularity, mesangial thickening, and congestion in the Bowman space in the kidney (Jin et al., 2015b).

Through comparison of viremia, hematological and biochemical parameters, and immune responses, rhesus macaques challenged with SFTSV show a number of key features of mild cases in humans. The reason for the variations between rhesus macaques and cynomolgus macaques remains unknown. It may be due to the different inoculation doses/routes or virus strains (SD4 vs. HB29), or genetic differences between primate species and needs to be further explored in the future.

### Cats

Recently, several studies showed that domestic animals especially cats may also play a significant role in SFTSV transmission to human as a potential source of infection (Park E. S. et al., 2019; Yamanaka et al., 2020; Ando et al., 2021), posing a much higher risk to their owners and the relevant practitioners and perhaps exacerbating the threat of SFTS to public health. Interestingly, cats (Breed: Russian Blue, American Shorthair) have been demonstrated to be highly susceptible to SFTSV in laboratory, and the severity of the infection seems independent of their age (Park E. S. et al., 2019; Figures 1, 2 and Table 1). In Park’s research, 4 of 6 cats succumbed to the infection of SFTSV (SPL010 strain, 10^7^ TCID_50_ by i.v. route) and all the infected cats showed similar symptoms with human patients, such as fever, leukocytopenia, thrombocytopenia, weight loss, anorexia, jaundice, and depression (Park E. S. et al., 2019). Moreover, in all existing SFTSV models, the cats are notable, considering the evident hemophagocytosis and the severe gastrointestinal tract injury very similar to but more severe than those in SFTS patients. The pathological lesions of fatal cats were characterized by hemorrhage in gastrointestinal mucosa, severe necrosis, hemorrhage and inflammatory infiltration in lymphatic tissues including spleen and lymph nodes, and notable hemophagocytosis in the spleen, bone marrow, lymph nodes and liver (Park E. S. et al., 2019). High level of viral titers was examined in blood, saliva, and tears, supporting that the companion animal might pose a novel potential threat to humans in SFTSV transmission by contacts of body fluids. Additionally, under electron microscopy, virions were observed in cytoplasmic vacuoles of immunoblast-like cells in the necrotizing cervical lymph nodes, indicating that immature B cells might be target cells of SFTSV (Park E. S. et al., 2019). Overall, the cats might be a much promising animal model as its high susceptibility and the clinical and histopathological manifestations like human cases of serious conditions. However, there are still many of hurdles to overcome. For example, cats are extremely hard to handle in biosafety level 3 (BSL-3) containment laboratories and the cost of experiments using cats is much higher, compared with routine laboratory rodents. Moreover, the experimental standard cat strains have not been cultivated yet. There might be great variations between different strains of cats, rendering the experimental results with cats not widely accepted.

## Conclusion

Outbreaks of emerging viral infectious diseases come with enormous social and economic burdens and challenges to public healthcare systems. SFTS is an emerging bunyaviral disease that is a severe threat around the world with no effective treatments or vaccines, and causes widespread transmission, severe symptoms, and high mortality. Creating suitable animal models that can accurately duplicate the hallmarks of human infection becomes non-negligible and prerequisite to lucubrate pathogenic mechanisms of SFTSV and set up the rapid and economical platform to evaluate safety and efficacy of antiviral drugs, vaccine candidates, or other treatment strategies prior to clinical trials in humans. As discussed in this review, multiple lethal or non-lethal animal models have been tested in varying degrees, providing potential options for *in vivo* investigations of SFTSV infection (Figure 1).

The following might be suggested to be taken seriously in SFTSV animal experiments: (1) the objective of the research should be certainly considered a top priority. For example, in order to elucidate viral pathogenesis, the model should reproduce representative aspects of the disease. As for efficacy evaluation of therapies, the viral replication, pathological processes, clinical manifestations, and outcomes of the SFTSV-infected animal models had better be tracked easily. (2) The next is to choose the corresponding lethal or non-lethal models with appropriate animal species and strains based on the research objectives. As aforementioned, there are huge differences existing in diverse animals with different genetic backgrounds and physiologic and immune conditions when infected with SFTSV, leading to differential susceptibility and various pathological manifestations. In addition, practicability, repeatability, and availability of animals are also important. Thus, standardized experimental animals, especially mice, are often chosen as a priority instead of the animals that have a gap in the quality control standards. (3) Note the differences in SFTSV strains and select the strains for animal infection experiments with caution. As reported, a series of SFTSV subtypes and strains are significantly different in virulence and pathogenicity in the aged ferret model (Yun et al., 2020). (4) Consider the influence of different infection methods (especially infection doses or routes) on outcomes of animal. The mortality and the severity of clinical symptoms can be SFTSV dose-dependent and vary with the routes of infection (Chen et al., 2012; Jin et al., 2012; Matsuno et al., 2017; Park et al., 2020). For example, in newborn mice, the group infected by i.c. had a higher mortality rate than the group infected by i.p. even at the same dosage (Chen et al., 2012). (5) Interpret the results fastidiously by closely referring to clinical data from human cases and correlate the valid data from animal experiments cautiously with those in human infections. Although the inherent differences between species cannot be overlooked, grasping the aspects of animal models similar to humans is a key objective of laboratory animal experiments. The animal experiments should serve for elimination of infection and pathogenesis in humans and be analyzed in conjunction with clinical findings in human cases.

To date, significant progress has been made in the studies on animal models of SFTSV infection. However, many limitations of the existing models remain apparent as pointed in the sections above. There is still an urgent need to establish more appropriate animal models and experimental methods used broadly or used for some specific studies. Obviously, it is challenging. Plenty of obstacles have to be overcome for future development and optimization of the animal infection models. Importantly, the mechanisms of SFTSV replication and pathogenesis in human are yet to be further delineated. Experimental studies based on human cell systems and clinical data from SFTS patients are both crucial for better understanding of the molecular mechanisms underlying viral infection and pathogenicity and for further development of animal models that are aimed to recapitulate the infection process in humans. In this sense, in-depth elucidation of SFTSV-host interactions will be informative. Particularly, knowledge of host antiviral or pro-viral factors and their functions and mechanisms in virus-host interplays may provide critical clues for creative development of animal models with specific gene deleted, modified, or humanized. As seen in the previous studies, STAT2 knockout and MOV10 knockdown both increased SFTSV replication and pathogenicity in adult mice, albeit to different extents. However, the consequences of STAT2 deletion are comprehensive and non-specific as it abolishes the IFN responses which have broad antiviral and immune-regulatory effects. In comparison, deficiency of a host restriction factor like MOV10 that directly targets the viral N protein, blocking RNP assembly and hence virus replication independently of the IFN antiviral system may have the advantages of minimal influence to the host immunity and specific increase of susceptibility to the viral infection. Unfortunately, MOV10 is required for mouse development and thus complete knockout of MOV10 results in embryonic death (Skariah et al., 2017). It will be warranted to further investigate new host factors significantly restricting or bolstering SFTSV infection or other approaches for gene engineering of animals. For example, as discussed in the section “*Stat2*^–/–^ and *Stat1*^–/–^ Mice,” mice with STAT2 humanized (rather than simply deleted) would be an interesting option for further exploration. On the other hand, animal-adapted strains of SFTSV may be worth a careful try of screening in biosafety laboratories in the future. Compared to the modification of animal models, screening and identification of adaptive mutant strains may be more readily achieved, as in many previous studies on other high-pathogenic viruses (Bray et al., 1998; Roberts et al., 2007; Gu et al., 2020; Guo et al., 2021; Hawman et al., 2021). Experiments of animal model infection with adapted strains would be competent for some researches.

## Author Contributions

HW, XS, FD, and Y-JN contributed to project administration and supervision. Y-JN and HW contributed to funding acquisition. Y-JN contributed to conceptualization and review and revision of the manuscript. JS, Y-QM, and YL contributed to literature search and did the comprehensive literature search using engines/databases like PubMed, Embase, Web of Science, Google Scholar, and Chinese National Knowledge Infrastructure (CNKI) with keywords including SFTSV (or the full name), huaiyangshan virus, phlebovirus, banyangvirus, bandavirus, bunyavirus, animal model, vaccine, antiviral drug, etc. JS, Y-QM, and Y-JN contributed to writing and editing of the original draft and reviewed and abstracted data from the related publications. Y-JN and JS contributed to visualization and created the figures accordingly. All authors read and approved the manuscript.

## Conflict of Interest

The authors declare that the research was conducted in the absence of any commercial or financial relationships that could be construed as a potential conflict of interest.

## Publisher’s Note

All claims expressed in this article are solely those of the authors and do not necessarily represent those of their affiliated organizations, or those of the publisher, the editors and the reviewers. Any product that may be evaluated in this article, or claim that may be made by its manufacturer, is not guaranteed or endorsed by the publisher.
